# Temporal gut microbiota dysbiosis links metabolic impairment, LDL desialylation, and accelerated atherosclerosis in LDLR^−^/^−^ mice

**DOI:** 10.3389/fmed.2026.1754833

**Published:** 2026-07-01

**Authors:** Guoying Guan, Wei Zhang, Yingying Zhuang, Jia Sheng, Siyi Wang, Nishang Zheng, Xinyue Wang, Hongwei Li, Yuhong Wang

**Affiliations:** 1 Department of General Practice, The First Affiliated Hospital of Harbin Medical University, Harbin, China; 2 State Key Laboratory of Vaccines for Infectious Diseases, Xiang An Biomedicine Laboratory, National Innovation Platform for Industry-Education Integration in Vaccine Research, School of Public Health, Xiamen University, Xiamen, China; 3 Clinical Nutrition Department, Zhongshan Hospital (Xiamen), Fudan University, Xiamen, China; 4 Xiamen Clinical Research Center for Cancer Therapy, Xiamen, China; 5 Xiamen Key Laboratory of Biotherapy, Xiamen, China; 6 Department of Geriatrics, The First Affiliated Hospital of Harbin Medical University, Harbin, China

**Keywords:** atherosclerosis, gut microbiota, high-cholesterol diet, metabolomics, sialic acid content on LDL

## Abstract

**Introduction:**

This study used low-density lipoprotein receptor-deficient (LDLR−/−) mice fed a high-cholesterol diet (HCD) to establish an accelerated atherosclerosis model and to characterize the temporal dynamics of gut microbiota remodeling during disease progression.

**Methods:**

Male C57BL/6J wild-type and LDLR−/− mice were fed either a normal diet (ND) or HCD for 12 weeks, with gut microbiota profiled at weeks 0, 8, and 12. HCD markedly accelerated atherogenesis in genetically susceptible mice, as evidenced by increased body weight, visceral adiposity, aggravated atherosclerotic lesions, and dyslipidemia.

**Results:**

Time-resolved microbiota analysis revealed progressive community reorganization, characterized by expansion of Bacillota and Actinomycetota and depletion of Bacteroidota, with the most pronounced alterations observed in the HCD group. These microbial shifts were closely associated with reduced sialic acid content on LDL particles, suggesting a potential link between gut microbial dysbiosis and LDL desialylation. Functional profiling further showed a dynamic transition in microbial metabolic capacity. At week 8, the HCD group exhibited a compensatory enhancement of selected metabolic pathways, whereas by week 12, broad functional deterioration emerged, involving energy metabolism, cellular structural biosynthesis, and genetic information-processing pathways. These findings indicate a progressive loss of microbial functional resilience under sustained high-cholesterol exposure. Metabolomic analysis revealed suppression of cholesterol metabolism and bile acid biosynthesis pathways, activation of insulin resistance-related signaling, and accumulation of candidate pathogenic metabolites, including specific diglycerides and 25-hydroxycholecalciferol. These metabolites were associated with enhanced inflammatory activation and reduced LDL sialylation, suggesting that diet-induced microbial and metabolic perturbations may converge to amplify systemic chronic inflammation and accelerate atherogenesis.

**Discussion:**

HCD promotes atherosclerosis progression in the context of LDLR deficiency by inducing time-dependent gut microbiota dysbiosis, characterized by structural reorganization and progressive functional impairment. This dysbiotic trajectory promotes pathogenic metabolite accumulation, systemic inflammatory activation, and LDL desialylation through a microbiota-metabolism-immune axis. These findings provide mechanistic insight into the gut microbial regulation of atherosclerosis and support the development of microecology-based strategies for cardiovascular disease prevention and intervention.

## Graphical Abstract

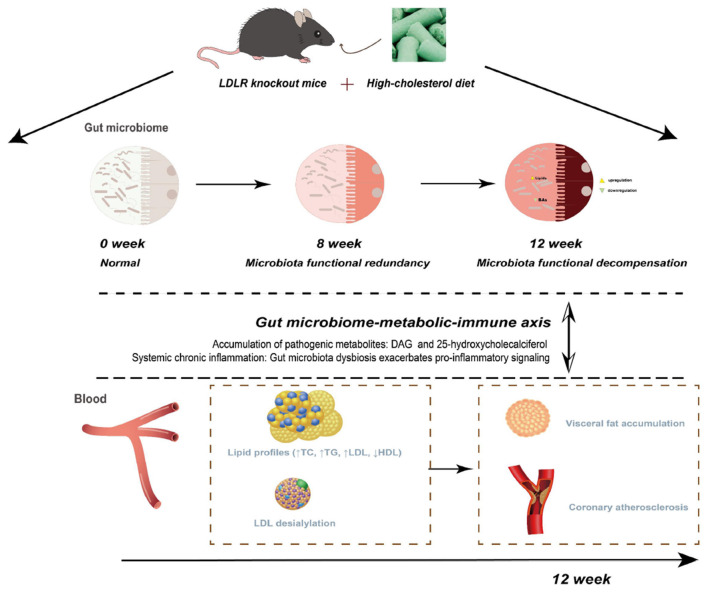
A graphical abstract. A high-cholesterol diet induces structural and functional dysregulation of the gut microbiota, which in turn activates the gut microbiome-metabolic-immune axis. This leads to accumulation of pathogenic metabolites [diacylglycerol (DGA) and 25-hydroxycholecalciferol] and systemic chronic inflammation. These changes ultimately accelerate coronary atherosclerosis progression by reducing sialic acid content on low-density lipoprotein (LDL-SIA), accompanied by dyslipidemia [elevated total cholesterol (TC), triglycerides (TG), and low-density lipoprotein (LDL); decreased high-density lipoprotein (HDL)] and visceral fat accumulation in low-density lipoprotein receptor knockout (LDLR−/−) mice.

## 1 Introduction

Atherosclerosis is a chronic inflammatory disease and a major driver of cardiovascular diseases (CVDs), ranking among the leading causes of death worldwide (1, 2). Although advances in modern medicine have significantly increased human life expectancy, the incidence of various chronic diseases, including atherosclerosis, continues to rise in the context of an aging society (3, 4).

The gut microbiota plays a crucial role in regulating host cardiovascular metabolism (5, 6). Gut dysbiosis—an imbalance in its composition and function—is closely associated with the development of numerous diseases, including atherosclerosis. Significant differences exist in the gut microbial structure between healthy and diseased states, with diet being one of the key factors influencing its composition. Dysbiosis can promote atherosclerosis through multiple mechanisms, such as by producing pro-inflammatory metabolites or activating host immune responses, thereby exacerbating systemic inflammation (7, 8). Gut microbes metabolize dietary components to generate various small-molecule metabolites, such as trimethylamine (TMA) and short-chain fatty acids, which play a central role in host-microbe interactions, influencing immune homeostasis and promoting inflammatory responses (7, 9). Furthermore, dysbiosis is often accompanied by impaired intestinal barrier function, leading to increased gut permeability. This allows harmful substances like lipopolysaccharide (LPS) and other endotoxins to translocate from the gut lumen into systemic circulation, triggering systemic inflammation and oxidative stress, thereby accelerating the development of atherosclerosis (10, 11). Additionally, the gut microbiota is involved in regulating lipid metabolism pathways, including bile acid and steroid metabolism. Dysbiosis can alter the bile acid profile, further disrupting cholesterol absorption and excretion, and promoting atherosclerosis (12).

Tracking the dynamic changes in the gut microbiota during the progression of atherosclerosis helps identify key microbial communities or specific bacterial species associated with different disease stages. Specific alterations in the gut microbiota and their metabolites have the potential to serve as biomarkers for early diagnosis or disease progression monitoring. A deeper understanding of the mechanisms by which the gut microbiota influences atherosclerosis will provide a theoretical basis for developing microbiota-targeted therapeutic strategies (12, 13).

Mouse models are indispensable in atherosclerosis research. Commonly used models, such as apolipoprotein E knockout (ApoE−/−) and low-density lipoprotein receptor knockout (LDLR−/−) mice, effectively mimic the pathological processes of human atherosclerosis, providing a valuable platform for investigating disease mechanisms, elucidating the role of the gut microbiota, and evaluating potential therapies (14, 15). This study utilizes LDLR−/− mice fed a high-cholesterol diet to establish an atherosclerosis model. By dynamically monitoring the changes in the gut microbiota during disease development, we aim to systematically reveal the temporal relationship between microbial succession and disease progression. The inclusion of wild-type mice as controls provides an internal reference for the genetic defect models, helping to distinguish the independent contributions of host genotype and gut microbiota changes to atherosclerosis development, thereby enhancing the reliability and specificity of the findings. Integrated metabolomic analysis enables the systematic identification of metabolites produced by the gut microbiota that participate in host physiological and pathological regulation, thereby providing deeper insights into their functional mechanisms in the disease process (12, 13). This comprehensive research strategy not only helps clarify the specific role of the gut microbiota in atherosclerosis but also holds promise for identifying potential therapeutic targets, offering a scientific basis for developing novel microecology-based interventions for the prevention and treatment of atherosclerosis.

## 2 Materials and methods

### 2.1 Animal experiments

Male C57BL/6J mice (*n* = 10) and LDLR−/− mice (*n* = 20) on a C57BL/6J background were obtained from Cyagen Biosciences (Suzhou) Co., Ltd. Animals were housed under standardized conditions (22 ± 1 °C, 40–60% relative humidity) with 12/12-h light-dark cycles and *ad libitum* access to water. Following a 1-week acclimation period, LDLR−/− mice were stratified into two dietary groups: Control model group (*n* = 10 LDLR−/− C57BL/6J background mice and *n* = 10 C57BL/6J mice): Maintained on standard chow [normal diet (ND), Beijing Keao Xieli Feed Co., Ltd.; Beijing Feed Certificate (2018) 0673)]. Experimental model group (*n* = 10 LDLR−/− mice): Fed a high-cholesterol diet (D12108C; Research Diets, Inc.) formulated with 40% fat-derived calories and 1.25% (w/w) cholesterol [Jiangsu Collaborative Pharmaceutical Bioengineering Co., Ltd., Su Feed Certificate (2019) 01008].

### 2.2 Ethical approval statement

All experimental procedures were performed in accordance with the guidelines of the Institutional Animal Care and Use Committee of the Laboratory Animal Center of Xia-men University and the International Association of Veterinary Editors guidelines for the Care and Use of Laboratory Animals. The protocols for animal use were reviewed and approved by the Animal Ethical and Welfare Committee of the Laboratory Animal Center of Xiamen University (Approval No. XMULAC20240172).

### 2.3 Sample collecting and index testing

Body weight and food intake were recorded weekly. Fasting blood glucose (FBG) was measured at weeks 0, 4, 8, and 10. After 10 weeks of intervention, mice were fasted for 12 h and subjected to an oral glucose tolerance test (OGTT) by gavage with 20% glucose solution (10 μL/g body weight). Blood glucose levels were measured from the tail vein at 0, 30, 60, and 120 min post-administration using a glucometer (Sanuo Biological Sensing Co., Ltd., China).

Following the 10-week intervention, mice were fasted for 12 h and anesthetized with 4% isoflurane (Shenzhen Reward Life Technology Co., Ltd., China), followed by euthanasia via cervical dislocation. Blood was collected from the retro-orbital plexus. Coronary artery tissues and colonic mucosa were promptly excised and rinsed with ice-cold saline (0.9% NaCl). Adipose tissues—including mesenteric, peritesticular, and perirenal fat—were dissected, rinsed, gently blotted, and weighed using an analytical balance (±0.1 mg). Blood samples were centrifuged at 2,000 rpm (382 × g, 4 °C) for 15 min to obtain serum, which was aliquoted for subsequent analysis. All specimens were snap-frozen in liquid nitrogen and stored at −80 C until further use.

### 2.4 Serum biochemical indicators

Serum concentrations of total cholesterol (TC), triglycerides (TG), HDL-C, and LDL-C were determined using an automated biochemistry analyzer (Mindray BS-220). Levels of serum oxidized LDL (ox-LDL) and coronary tissue cytokines/chemokines (CCL5, CXCL1, IL-1, IL-2, IFN-γ, LOX1) were measured with corresponding species-specific ELISA kits (Wuhan Beinle Biotechnology Co., Ltd., China), following the manufacturer's instructions.

Sialic acid content on LDL (LDL-SIA): the fresh plasma was transferred into a centrifuge tube, adjusted to a density of 1.006 g/mL, and sealed with nitrogen. After centrifugation at 40,000 r/min for 24 h at 10 C, the upper layer of liquid was collected to obtain very low density lipoprotein (VLDL); the lower layer of liquid was adjusted to 1.063 g/mL, and then centrifuged at 40,000 r/min for 24 h at 10 C to obtain LDL; the lipoproteins were dialyzed, and then filled with nitrogen and sealed for later use.

The protein concentration of the isolated LDL was determined using the bicinchoninic acid (BCA) assay (Thermo Fisher Scientific, USA). An aliquot containing 50 μg of protein was transferred into a 5 mL centrifuge tube and centrifuged at 4,500 r/min for 10 min. After adding 1 mL of 0.1 mol/L sulfuric acid, the sample was placed in a water bath at 80 °C for 2 h, then removed and cooled down. An equal volume of 10% trichloroacetic acid (TCA) was added and the mixture was mixed well and left for 10 min at 4 C. Subsequently, the mixture was left for an additional 10 min and then centrifuged at 4,500 r/min for 10 min. The resulting supernatant was transferred to a new tube. Cold 5% TCA was added to the precipitate and mixed well. The mixture was centrifuged again at 4,500 r/min for 10 min, and the two supernatants were combined. The combined supernatant was then filtered before further analysis. Remove, cool down, and then filter through a 0.22 microporous filter membrane. The filtrate (0.1 mL) was directly used for ultra-performance liquid chromatography-tandem mass spectrometry (UPLC-MS/MS) analysis without a derivatization step, because mass spectrometry detects native sialic acid.

Sialic acid content was measured using an UPLC-MS/MS system consisting of a Waters UPLC system and an AB Sciex 4000 QTRAP MS/MS system. Chromatographic separation was performed on a Waters HSS T3 column (2.1 mm × 100 mm, 1.8 μm particle size) maintained at 30 °C. The autosampler temperature was set at 10 °C, and the flow rate was 0.3 mL/min. Mobile phase A was ultrapure water and mobile phase B was acetonitrile. The gradient elution program was as follows: 0–0.5 min, 95% A and 5% B; 1.0 min, 40% A and 60% B; 3.0 min, 5% A and 95% B; 4.0 min, 95% A and 5% B; and held at 95% A and 5% B until 5.0 min. The total run time was 5.0 min, and the injection volume was 10 μL. Mass spectrometry was performed in electrospray ionization negative mode (ESI?) with an ion spray voltage of −4,500 V and an ion source temperature of 450 °C. The curtain gas (CUR), collision gas (CAD), ion source gas 1 (GS1), and ion source gas 2 (GS2) were set at 35, 7, 40, and 50 arb, respectively. Multiple reaction monitoring (MRM) was used with the quantitative ion pair m/z 308.2500 → 87.1000 and the qualitative ion pair m/z 308.2500 → 170.2500, respectively, which are consistent with previously reported LC-MS/MS methods (16, 17). For method validation, a calibration curve was constructed using sialic acid standards (10–1,000 ng/mL; The linear regression equation was y = 786.036x + 0.000, with a coefficient of determination *R*^2^ = 0.9999 (Supplementary Table S1). Sialic acid content was normalized to protein concentration, and expressed as μg sialic acid per mg protein. The signal-to-noise (S/N) ratio for the lowest calibrator was 29.47. Peak purity assessment was not applicable because MRM detection is highly specific; instead, the ratio of the two MRM transitions (qualifier/quantifier) was monitored to confirm peak identity, with an acceptable tolerance of ±20%. All samples met this criterion.

### 2.5 Gross oil red staining and H&E staining in coronary

Gross Oil Red O Staining of Coronary Tissues: Isolated coronary vessels were fixed in 4% paraformaldehyde for over 24 h, washed with PBS, and carefully dissected longitudinally. The tissues were briefly rinsed with tap water, sequentially immersed in 60% isopropyl alcohol and Oil Red O solution (Solarbio Life Sciences, Beijing, China), and incubated at 37 C in the dark for 60 min. After differentiation in 60% isopropyl alcohol until atherosclerotic plaques were distinctly red against a colorless background, samples were rinsed with distilled water, dried, and photographed on a scaled plate under optimized microscopy settings (Leica DM4B, Germany).

Histological Staining: For section-based analysis, fixed tissues were paraffin-embedded and sectioned. Oil Red O staining was performed using a modified kit (Solarbio Life Sciences) according to the manufacturer's instructions. Consecutive sections were subjected to hematoxylin and eosin (H&E) staining using a commercial kit (Solarbio Life Sciences). All stained sections were evaluated under light microscopy.

### 2.6 Gut microbiome

Colon content samples were flash-frozen in liquid nitrogen and stored at −80 °C until analysis. Total genomic DNA was extracted using the FastPure Stool DNA Isolation Kit (MJYH, Shanghai, China). The V3–V4 hypervariable region of the bacterial 16S rRNA gene was amplified with barcoded primers 341F (CCTACGGGNGGCWGCAG) and 806R (GGACTACHVGGGTATCTAAT). PCR amplicons were purified from 2% agarose gels using a PCR Clean-Up Kit (YuHua, Shanghai, China), followed by quantification on a Qubit 4.0 (Thermo Fisher Scientific, USA). Libraries were constructed using the NEXTFLEX^®^ Rapid DNA-Seq Kit (Bioo Scientific, Austin, TX, USA) following the manufacturer's protocol, which included adapter ligation, bead-based size selection, and PCR enrichment. Paired-end sequencing (2 × 300 bp) was performed on an Illumina NextSeq 2000 platform (PE300) at Majorbio Bio-Pharm Technology Co., Ltd. (Shanghai, China). After demultiplexing, paired-end reads were quality-filtered with fastp (v0.19.6) (18) and assembled using FLASH (v1.2.11) (19). High-quality sequences were processed with DADA2 in QIIME2 (2020.2) (20) to generate amplicon sequence variants (ASVs). All samples were rarefied to 25,691 reads per sample, achieving 99.9% Good's coverage.- Taxonomy was assigned against the SILVA v138 database via a QIIME2 classifier (21), and functional potential was inferred using PICRUSt2 (22) with HMMER, EPA-NG, castor, and MinPath. Subsequent analyses of community composition, diversity, and predicted function were performed on the Majorbio Cloud Platform (https://cloud.majorbio.com). The population grouping was performed at the ASV level (amplicon sequence variant level). Each ASV was treated as a distinct microbial taxon, and its prevalence (detection rate) across all samples within a given group was calculated to classify it into one of three categories:

Transient species: low prevalence (prevalence < 0.2); Intermediate species: moderate prevalence (between 0.2 and 0.8); Persistent (core) species: high prevalence (prevalence > 0.8). These thresholds are commonly used in microbial ecology studies to define core vs. transient taxa. The grouping criteria aim to distinguish environmentally sensitive (transient) from stable (persistent) microbial populations based on their detection frequency across samples (23).

### 2.7 Metabolomics analysis of the contents of colon

Mouse tissue samples were preprocessed as previously described (24). LC-MS/MS analysis was performed on a SCIEX UPLC-Triple TOF 6600 system system equipped with an ACQUITY HSS T3 column (100 mm × 2.1 mm, 1.8 μm; Waters, USA) at Majorbio Bio-Pharm Technology Co., Ltd. (Shanghai, China). The mobile phase consisted of (A) 0.1% formic acid in water:acetonitrile (95:5, v/v) and (B) 0.1% formic acid in acetonitrile:isopropanol:water (47.5:47.5:5, v/v), with a flow rate of 0.40 mL/min and column temperature maintained at 45 °C. The mass spectrometer was operated in both positive and negative electrospray ionization modes. The optimal MS conditions were set as follows (25): source temperature at 500 °C; sheath gas flow rate at 50 psi; aux gas flow rate at 13 arb; curtain gas flow rate at 35 psi; ion-spray voltage floating (ISVF) at 5,500 V in positive mode and −4,500 V in negative mode; normalized collision energy rolling from 20 to 40 to 60 V for MS/MS. Data were acquired using data-dependent acquisition (DDA) over a mass range of 50–1,200 m/z.

To monitor analytical stability, a pooled quality control (QC) sample was prepared by mixing equal volumes of all study samples (26). The QC sample was processed and analyzed identically to the experimental samples and was injected at regular intervals (every 6 samples). Raw LC-MS data were processed using Progenesis QI software (Waters, USA) to generate a three-dimensional data matrix in CSV format. Metabolite identification was conducted by querying the HMDB, Metlin, and Majorbio databases. The resulting data matrix was subsequently uploaded to the Majorbio Cloud Platform (https://cloud.majorbio.com) for further bioinformatic analysis. Data preprocessing on the platform included: retaining metabolic features detected in at least 80% of samples within any group; filling missing values with the minimum value; performing sum normalization to correct for sample preparation and instrument variation; excluding variables with relative standard deviation (RSD) > 30% in QC samples; and applying log10 transformation (27). The normalized data matrix was then used for subsequent multivariate analysis and differential metabolite screening.

### 2.8 Statistical analysis

All continuous variables were assessed for normality using the Shapiro-Wilk test and for homogeneity of variances using Levene's test (α = 0.05). Based on these assessments, data were analyzed as follows: one-way ANOVA with Fisher's LSD *post hoc* test for data meeting both assumptions; Welch's ANOVA with Dunnett's T3 test for normally distributed data with heteroscedasticity; and the Kruskal-Wallis *H*-test with Nemenyi-Damico-Wolfe-Dunn *post-hoc* analysis for non-parametric data. For multivariate repeated measures, a linear mixed-effects model was fitted using the Kenward-Roger approximation. All analyses were conducted at a two-tailed significance level of α = 0.05, with *p*-values adjusted for multiple comparisons where applicable, using SPSS 22.0 (IBM Corp.).

## 3 Results

### 3.1 Body weight, food intake energy and visceral fat

**Figure 1:**
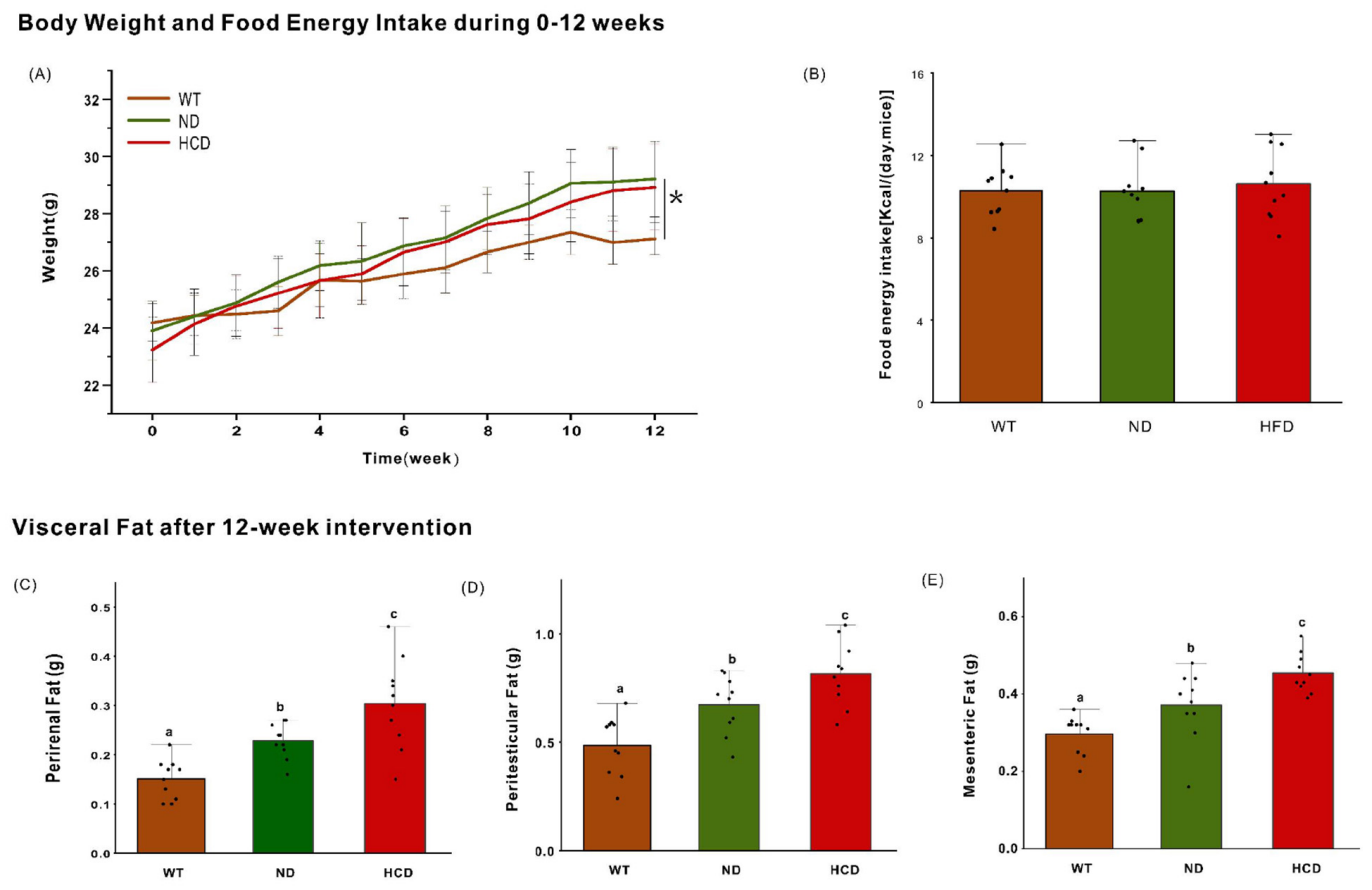
Body Weight, Food Intake Energy and Visceral Fat. **(A)** Body weight during 0–12 week; **(B)** Average daily food energy intake per mouse; **(C–E)** Visceral fat after 12-week intervention: **(C)** Perirenal fat; **(D)** Peritesticular fat; **(E)** Mesenteric fat. The difference between values with completely different superscripts was statistically significant, *P* < 0.05. Data are means ± SD (*n* = 10). ^*^ denotes statistically significant differences between groups.

As shown in Figure 1, despite no significant difference in daily energy intake, both the ND and HCD groups exhibited a more rapid increase in body weight compared to the WT group. By the end of the intervention, both the ND and HCD groups showed significantly higher body weight and visceral adipose tissue (including perirenal, epididymal, and mesenteric fat) mass than the WT group (*P* < 0.05). Although there was no statistically significant difference in overall body weight between the ND and HCD groups, the HCD group had significantly higher body fat content than the ND group (*P* < 0.05). These findings suggest that a high-cholesterol diet may specifically promote fat accumulation, independent of total body weight changes, indicating that dietary components such as high cholesterol may directly modulate lipid metabolism and fat distribution patterns.

Further analysis revealed that LDLR−/− knockout mice, due to impaired LDL metabolic pathways, accumulated approximately 24.91%−50.99% more fat than wild-type mice, indicating that the genetic defect of LDLR deletion alone can independently induce lipid metabolism disorders and promote fat deposition. Under the combined influence of a high-cholesterol diet and genetic predisposition, fat accumulation in mice further increased by approximately 56.23%−101%, suggesting that in genetically susceptible individuals, a high-cholesterol diet can significantly amplify the effect of fat accumulation, thereby accelerating the progression of metabolic diseases.

### 3.2 Coronary pathology and core factors in atherosclerosis

**Figure 2:**
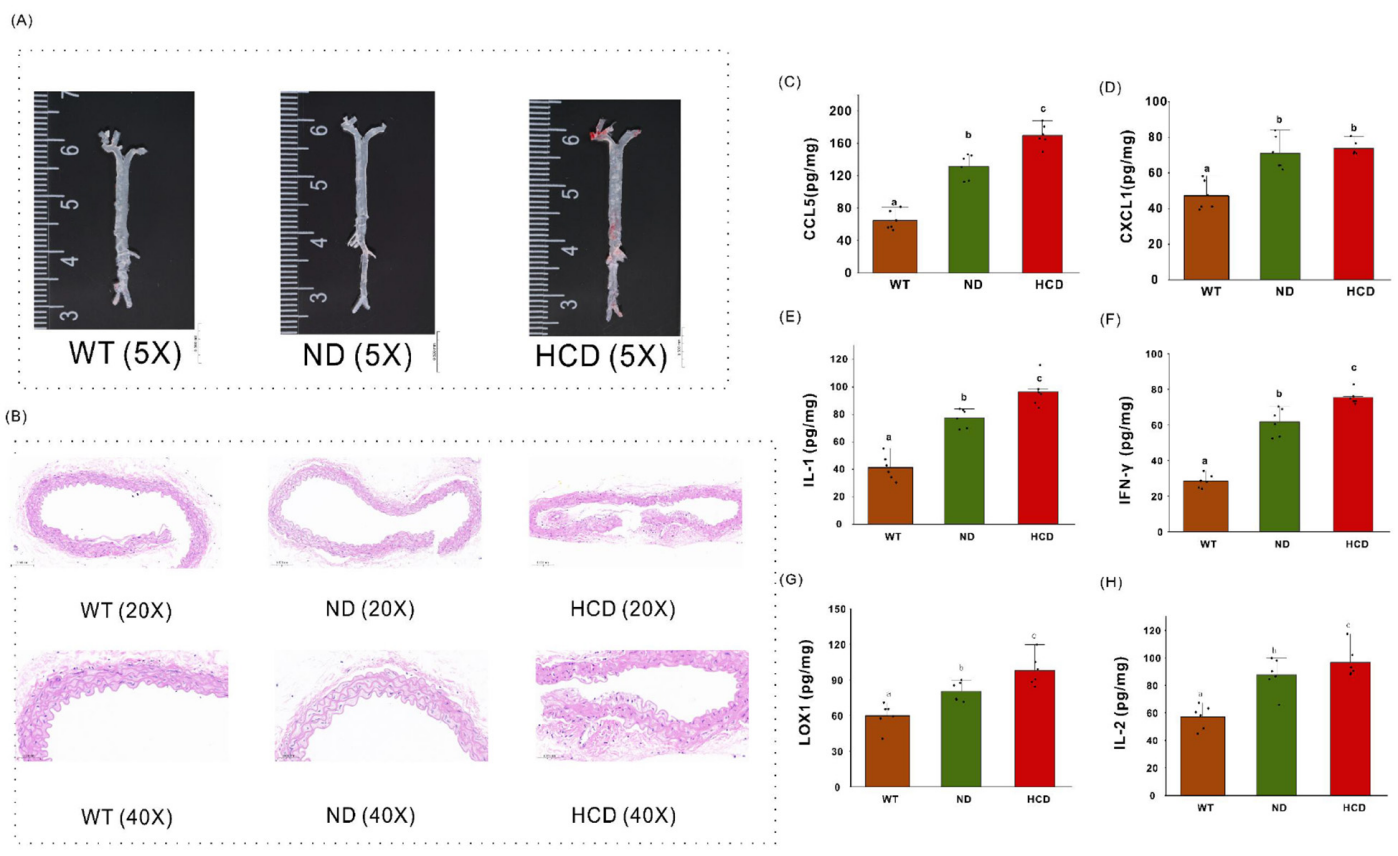
Coronary Pathology and Core Factors in Atherosclerosis After 12-week Intervention. **(A)** Gross Oil Red O Staining of Aortic Atherosclerotic Lesions; **(B)** Histological Analysis of Aortic Tissues by gross Oil Red O staining; **(C–H)** Key Atherosclerotic Metrics in Coronary Artery Tissues: **(C)** C-C Motif Chemokine Ligand 5 (CCL5); **(D)** C-X-C Motif Chemokine Ligand 1 (CXCL1); **(E)** Interleukin-1(IL-1); **(F)** Interferon Gamma (INF-γ); **(H)** Lectin-Type Oxidized LDL Receptor 1 (LOX1); **(H)** Interleukin-2 (IL-2). The difference between values with completely different superscripts was statistically significant, *PP* < 0.05. Data are means ± SD (*n* = 86).

As shown in Figure 2, both gross Oil Red O staining and H&E staining of the coronary arteries indicated that the high-cholesterol diet (HCD) group exhibited more severe coronary artery lesions compared to the normal diet (ND) and wild-type (WT) groups. In addition, the levels of CCL5, CXCL1, IL-1, IFN-γ, LOX1, and IL-2 in coronary artery tissues were significantly higher in the HCD group than in the ND and WT groups (*P* < 0.05). It is worth noting that although no significant morphological differences were observed in pathological sections between the ND and WT groups, the levels of key atherosclerosis-related factors (CCL5, CXCL1, IL-1, IFN-γ, LOX1, IL-2) in the coronary arteries of the ND group were generally higher than those in the WT group. These results suggest that a high-cholesterol diet can accelerate the development of atherosclerosis in genetically susceptible individuals, and even in the absence of significant pathological changes, genetically predisposed individuals may already be at a higher risk of developing the disease.

### 3.3 Glycolipid metabolism

**Figure 3:**
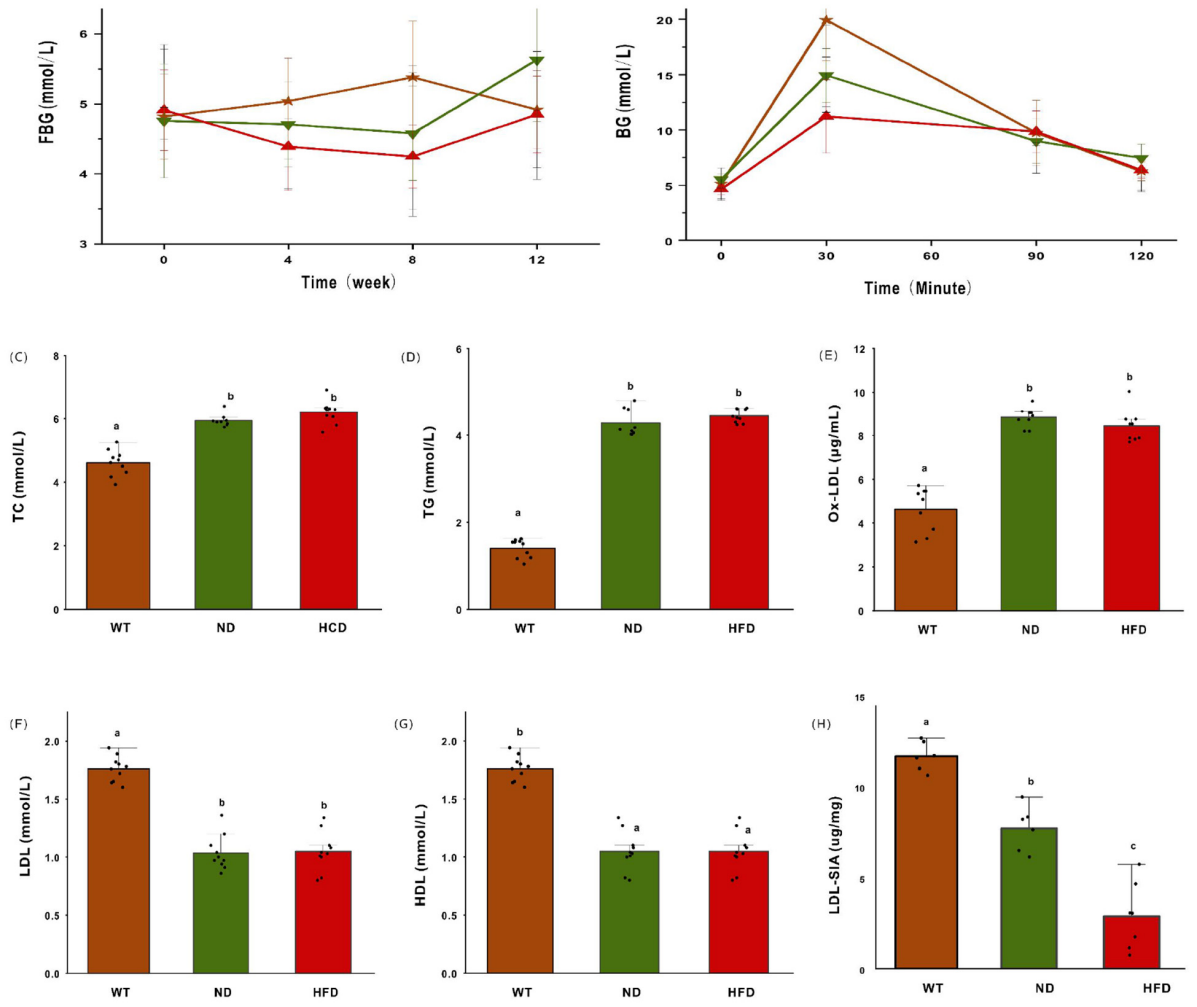
Glycolipid Metabolism After 12-week Intervention. **(A, B)** Glucose metabolism: **(A)** Fasting Blood Glucose (FBG); **(B)** Oral glucose tolerance tests (OGTT); **(C–H)** Lipid metabolism: **(C)** Total cholesterol (TC); **(D)** Triglycerides (TG); **(E)** Oxidized low-density lipoprotein (Ox-LDL) **(F)** Low-density lipoprotein (LDL); **(G)** High-density lipoprotein (HDL); **(H)** Low-density lipoprotein sialic acid content (LDL-SIA). The difference between values with completely different superscripts was statistically significant, *P* < 0.05. Data are means ± SD (*n* = 10).

As shown in Figures 3A, B, no significant differences were observed in the fasting blood glucose trends among the groups in terms of glucose metabolism (*P* > 0.05). However, the oral glucose tolerance test (OGTT) conducted after the intervention revealed that the ND and HCD groups exhibited lower increases in blood glucose, with lower peak levels and a slower decline, strongly indicating marked insulin resistance in these mice. This may be accompanied by early to mid-stage pancreatic β-cell dysfunction, leading to reduced insulin sensitivity in response to a glucose load.

As seen in Figures 3C–H, in terms of lipid metabolism, compared to the WT group, the ND and HCD groups showed elevated serum levels of triglycerides (TG), total cholesterol (TC), low-density lipoprotein (LDL), and oxidized low-density lipoprotein (Ox-LDL), while high-density lipoprotein (HDL) levels were reduced (*P* < 0.05). It is noteworthy that although no statistical differences were observed in the four lipid parameters and Ox-LDL levels between the ND and HCD groups, the LDL-SIA in the ND group was higher than that in the HCD group, with the WT group showing the highest level. Combined with the degree of coronary artery lesions, these findings suggest that LDL-SIA may serve as a novel core indicator for predicting atherosclerosis risk.

### 3.4 Gut microbiome

**Figure 4:**
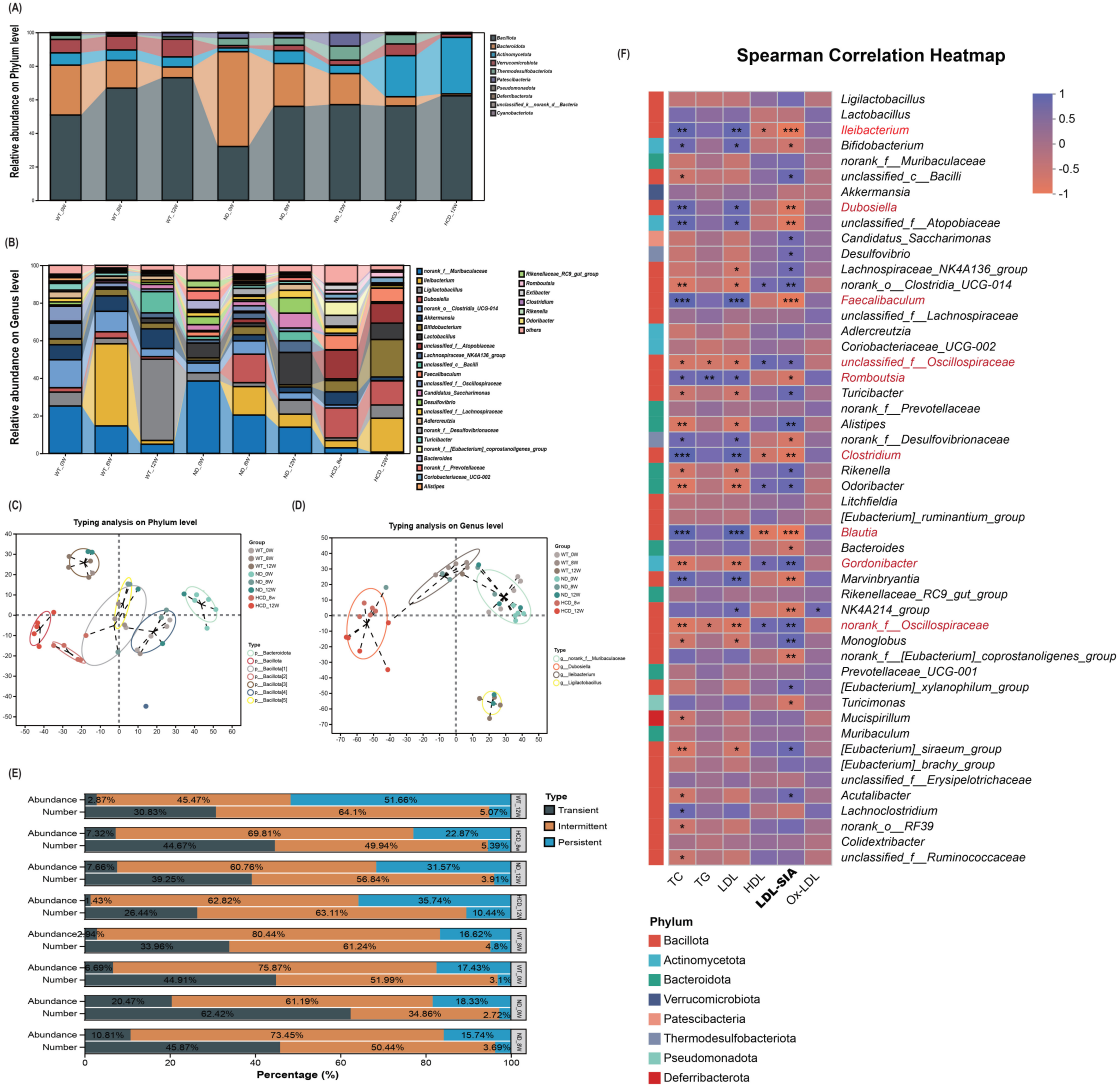
Gut Microbiome during 0–12 weeks intervention. **(A–D)** Species composition and diversity: **(A)** Relative abundance in phylum level; **(B)** Relative abundance in genus level; **(C)** Typing analysis on Phylum level of group samples relationships; **(D)** Typing analysis on Genus level of group samples relationships; **(E)** The composition of transient, intermittent, and persistent populations in the intestinal tract of each group at different time points; **(F)** Pearson Correlation Heatmap serum TC, TG, LDL, Ox-LDL, LDL-SIA and HDL with intestinal flora in Genus level. * denotes statistically significant differences between groups, * 0.01 < *P* ≤ 0.05, ** 0.001 < *P* ≤ 0.01, *** *P* ≤ 0.001.

As shown in Figures 4A, B, the composition of the gut microbiota in each group of mice at 0, 8, and 12 weeks exhibited certain patterns of change. At the phylum level, *Bacillota* and *Actinomycetota* showed an increasing trend in all groups, while *Bacteroidota* decreased. Specifically, *Bacillota* increased more rapidly in the WT group, *Actinomycetota* showed a more pronounced increase in the HCD group, and *Bacteroidota* and *Verrucomicrobiota* declined more sharply in the ND and HCD groups. At the genus level, *norank_f__Muribaculaceae* decreased across all groups. *Ileibacterium* displayed an initial increase followed by a decrease in the WT and ND groups, whereas in the HCD group, it first decreased and then increased. *Dubosiella, Bifidobacterium, unclassified_f__Atopobiaceae*, and *Faecalibaculum* showed significant increases in the HCD group, while *norank_o__Clostridia_UCG-014, Akkermansia, unclassified_f__Oscillospiraceae*, and *Turicibacter* exhibited declining trends.

Figures 4C–E reveal that the gut microbiota structure of each group clustered together during dynamic changes. Analysis of temporal retention patterns showed an overall increase in the proportion of persistent bacteria from week 0 to week 12 in all three diet groups. Notably, the HCD group exhibited a continuous, monotonic increase (from 18.33% to 22.87% to 35.74%), whereas the other groups showed a slight decrease at week 8 before increasing at week 12. This suggests that the HCD diet may drive the microbiota toward a more consistently stable configuration. To further validate that our persistent (core) species identified by prevalence > 0.8 also exhibit high average abundance, we performed linear regression between prevalence and log10-transformed mean relative abundance. The significant positive correlation (*R*^2^ = 0.718, *P* < 0.0001) supports the ecological relevance of our grouping criteria (Supplementary Figure S2A). After the intervention, correlations were observed between microbial abundance and serum LDL-SIA levels: increases in *Ileibacterium, Dubosiella*, and *Faecalibaculum* were associated with reduced LDL-SIA, while decreases in *norank_o__Clostridia_UCG-014, Turicibacter*, and *unclassified_f__Oscillospiraceae* were also linked to lower LDL-SIA (Figure 4F). In the HCD group, the rise in the first three genera and the decline in the latter three may collectively contribute to decreased LDL-SIA levels, thereby promoting the progression of atherosclerosis.

**Figure 5:**
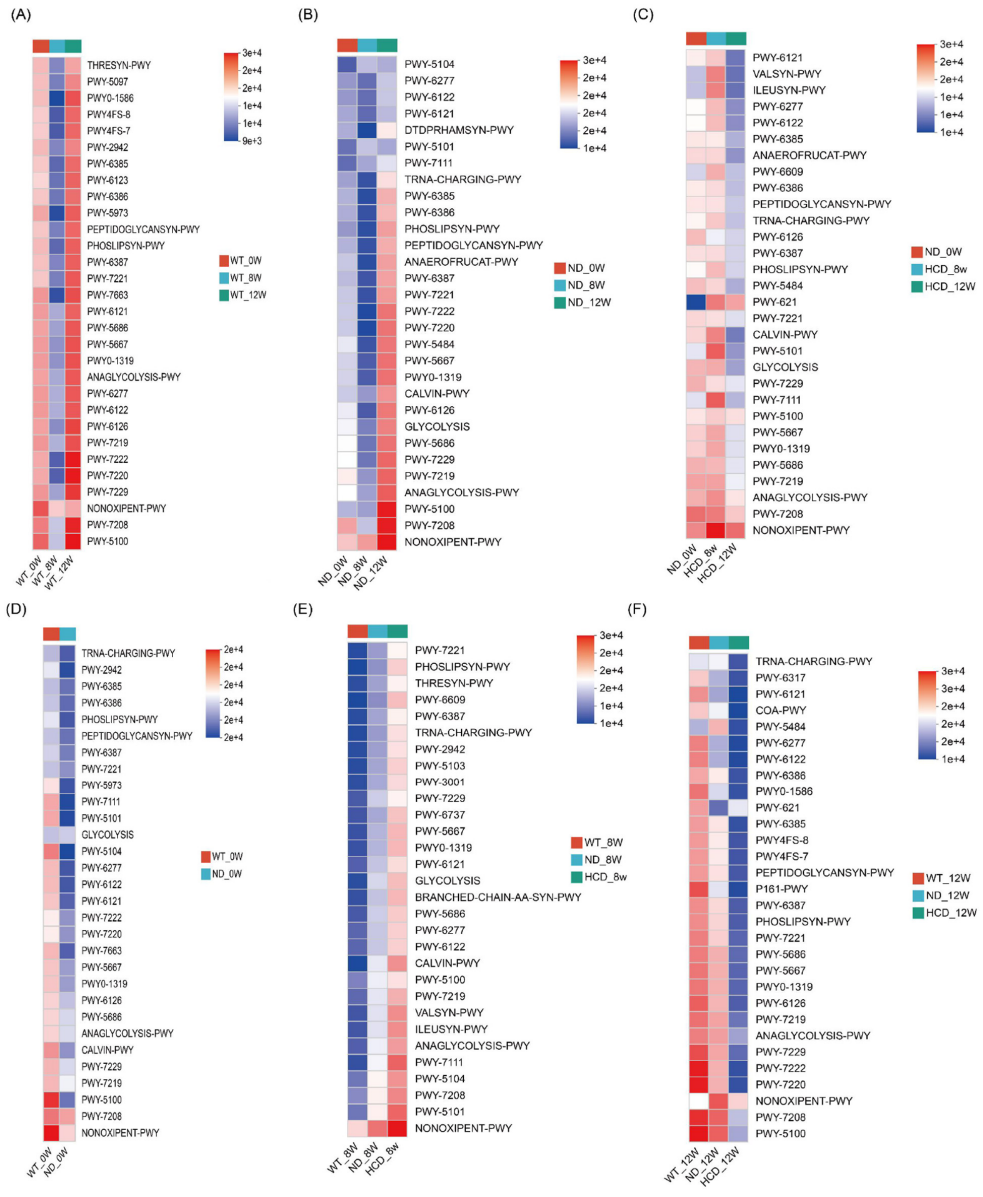
Functional Analysis of Flora After 12-week Intervention. **(A)** MetaCyc pathway enrichment analysis based on PICRUSt2 inference was performed on WT group mice at 0, 8, and 12 weeks; **(B)** MetaCyc pathway enrichment analysis based on PICRUSt2 inference was performed on ND group mice at 0, 8, and 12 weeks; **(C)** MetaCyc pathway enrichment analysis based on PICRUSt2 inference was performed on HCD group mice at 0, 8, and 12 weeks. **(D)** MetaCyc pathway enrichment analysis based on PICRUSt2 inference was performed on the three groups of mice at week 0; **(E)** MetaCyc pathway enrichment analysis based on PICRUSt2 inference was performed on the three groups of mice at week 8; **(F)** MetaCyc pathway enrichment analysis based on PICRUSt2 inference was performed on the three groups of mice at week 12. Note: The specific MetaCyc pathway that corresponds to each BioCyc ID can be found in the supplementary table titled “Supplementary materials-Drawing Pathway notes.”

Functional analysis of the gut microbiota is shown in Figure 5. In the WT group, from week 0 to week 12, microbial functions related to cell wall (peptidoglycan) and cell membrane (phospholipid) synthesis, provision of DNA and RNA building blocks (nucleotides), synthesis of essential small molecules (such as specific amino acids and fatty acids), and energy metabolism were enhanced. In the ND group over the same period, although energy metabolism, cellular structure construction, and genetic material replication functions improved, the synthesis of key growth factors (such as specific amino acids and nucleotide precursors) declined, indicating that bacterial growth rates may have exceeded the capacity of certain synthetic pathways. In contrast, the HCD group exhibited a comprehensive decline in energy metabolism, cellular structure construction, genetic material replication, and synthesis of specific amino acids, reflecting severe suppression of overall metabolic activity in the gut microbiota. The bacterial community may have entered a stagnant state resembling “dormancy” or “survival mode,” which is typically a marker of severe dysbiosis and indicates a deterioration in gut microecological health. Comparative analysis of the three groups further suggests that although gut microbiota function showed compensatory enhancement by the 8th week of HCD intervention, it progressed to severe functional imbalance by the 12th week.

To investigate which microbial populations are associated with the functional impairment in the HCD group, we correlated ASV abundances from each prevalence group with MetaCyc pathway abundances. Interestingly, only persistent (core) ASVs showed a notable proportion of significant correlations (8/47, 17.0%), whereas intermediate (5/175, 2.9%) and transient (0/86, 0%) ASVs exhibited very few or no significant associations (Supplementary Figures S2B–D and Supplementary Materials - Abundance table of transient, intermediate, and persistent ASVs). This pattern indicates that core microbiota members, despite their low numerical abundance as ASVs, are the primary functional drivers in the community. The lack of significant correlations for most intermediate and transient taxa may reflect their sporadic occurrence, functional redundancy, or condition-specific roles that are not captured by global correlation analysis across all samples.

Nevertheless, even among persistent ASVs, only 8 out of 47 reached statistical significance. This suggests that the pronounced functional decline in energy metabolism, cell wall biosynthesis, nucleotide metabolism, and amino acid synthesis (Figure 5) is not simply a linear consequence of the abundance of individual core taxa. Instead, it likely involves more complex mechanisms, including (i) community-wide compositional shifts that alter collective functional potential (28, 29), (ii) post-transcriptional regulation or metabolic dormancy of core species (i.e., the bacteria may be present but metabolically suppressed) (30), and (iii) functional redundancy that buffers against single-species fluctuations (28, 29). These findings highlight the importance of moving beyond single-taxon correlations when assessing microbiome functional impairment.

### 3.5 Metabolomic analysis of colonic content and its correlation with gut microbiota

**Figure 6:**
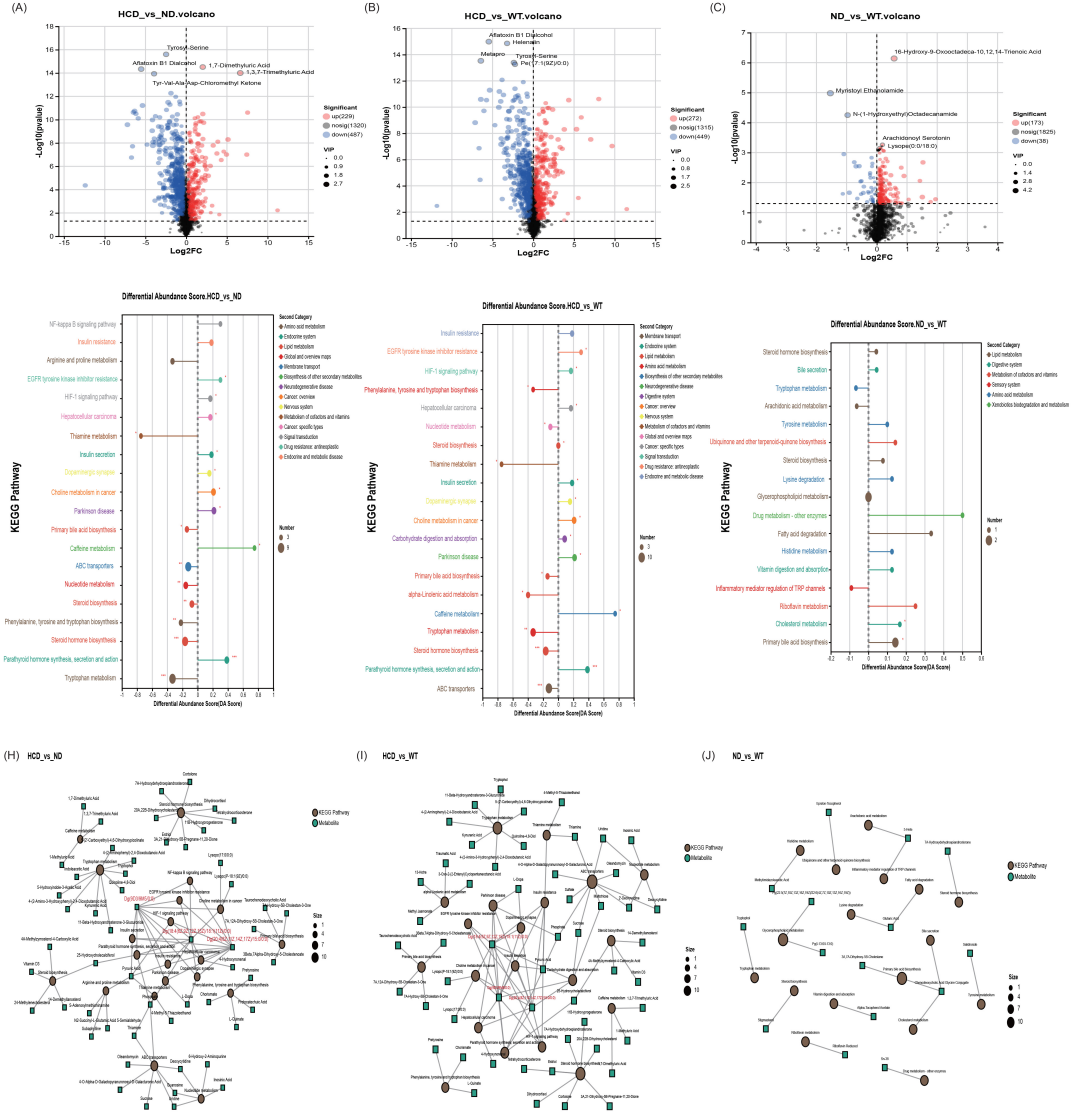
Comparison of metabolomic expression in colonic content after 12-week Intervention. **(A–C)** Differentially Metabolites: **(A)** Volcano plot comparing differential metabolites between the HCD and ND groups; **(B)** Volcano plot comparing differential metabolites between the HCD and WT groups; **(C)** Volcano plot comparing differential metabolites between the ND and WT groups; **(E–G)** Functional Enrichment in Colonic content: **(E)** Differential Abundance Score: TOP 20 KEGG pathways enriched with differential metabolites between HCD group and ND group; **(F)** Differential Abundance Score: TOP 20 KEGG pathways enriched with differential metabolites between HCD group and WT group; **(G)** Differential abundance score: TOP 20 KEGG pathways enriched with differential metabolites between ND group and WT group; **(H–J)** A network diagram displays the KEGG enrichment results for differential metabolites: **(H)** Network diagram of KEGG enrichment for differential metabolites (HCD vs. ND); **(I)** Network diagram of KEGG enrichment for differential metabolites (HCD vs. WT); **(J)** Network diagram of KEGG enrichment for differential metabolites (ND vs. WT).

As shown in Figure 6 and Figure S1, metabolomic analysis of colonic contents revealed that the HCD group exhibited a significantly greater number of differential metabolites compared to both the ND and WT groups, with approximately over 200 upregulated and over 400 downregulated metabolites. In contrast, the number of differential metabolites between the ND and WT groups was relatively small, with only 173 up-regulated and 38 down-regulated (Figure 6C). Functional enrichment analysis indicated that the high-cholesterol diet led to the downregulation of pathways related to amino acid synthesis and lipid metabolism—including cholesterol metabolism and bile acid synthesis—in LDLR−/− mice, while pathways associated with insulin resistance, endocrine and metabolic diseases, cancer, and neurodegenerative diseases were significantly enhanced.

A network diagram constructed based on differential metabolites and signaling pathways further identified three key differential metabolites: Dg(18:4(6Z,9Z,12Z,15Z)/18:1(11Z)/0:0), Dg(9D3/9M5/0:0), and Dg(20:4(8Z,11Z,14Z,17Z)/15:0/0:0). Correlation analysis showed that Dg(18:4(6Z,9Z,12Z,15Z)/18:1(11Z)/0:0), Dg(9D3/9M5/0:0), and 25-Hydroxycholecalciferol were negatively correlated with metabolites associated with cardiovascular protection, neuroregulation, and hormonal effects. In the HCD group, the levels of these four metabolites—Dg(18:4(6Z,9Z,12Z,15Z)/18:1(11Z)/0:0), Dg(9D3/9M5/0:0), 25-Hydroxycholecalciferol, and Dg(20:4(8Z,11Z,14Z,17Z)/15:0/0:0)—were significantly higher than those in the ND and WT groups, suggesting that these metabolites may serve as key metabolic markers driving disease progression induced by a high-cholesterol diet.

## 4 Discussion

This study successfully established an atherosclerosis progression model by intervening in LDLR knockout mice with a high-cholesterol diet. The results showed that the combined effect of dietary factors and genetic susceptibility doubled the degree of disease development compared to mice with a genetic background alone. This effect was closely related to significant alterations in the structure and function of the gut microbiota during atherosclerosis. It is particularly noteworthy that gut microbiota dysbiosis was potentially associated with a decrease in the sialylation level of serum lipoproteins, accompanied by changes in the intestinal metabolic profile, collectively exacerbating the pathological process of atherosclerosis.

Regarding the gut microbiota, dynamic analysis revealed that in high-cholesterol-fed LDLR−/− mice, the relative abundance of genera associated with inflammatory diseases, obesity, and high-fat diets, such as *Ileibacterium, Dubosiella*, and *Faecalibaculum* increased (31–33). In contrast, beneficial or commensal bacteria capable of producing anti-inflammatory substances like butyrate and strengthening the intestinal barrier, such as ^*^Clostridia_*UCG*−014^*^, *Akkermansia*, unclassified *Oscillospiraceae*, and *Turicibacter were significantly reduced* (32, 34–36). This shift in microbiota structure led to functional disruption of the gut microecology, exacerbating “pro-inflammatory signaling” and thereby promoting the activation of systemic inflammatory signals, resulting in significantly higher inflammation levels in the high-cholesterol group compared to the normal diet group. Research indicates that the intensified inflammatory state further upregulates sialidase expression, leading to an overall increase in systemic desialylation, which in turn further aggravates inflammation (37–39). This also explains why, despite comparable plasma lipid levels to the normal group, the high-cholesterol group exhibited significantly reduced sialylation of low-density lipoprotein (LDL). Existing studies point out that LDL desialylation is not only an independent risk factor for atherosclerosis (40, 41) but also a precursor to the formation of oxidized LDL (Ox-LDL)—desialylated LDL is more readily oxidized into Ox-LDL (42) and more easily taken up by phagocytic cells within the inflamed arterial wall, thereby promoting lipid deposition and atherosclerotic plaque formation (42, 43). This mechanism further elucidates the more severe atherosclerotic lesions observed in the high-cholesterol group animals compared to the normal group.

In the normal diet group, lacking the strong perturbation of a high-fat diet, the gut microbiota, although changing with age, exhibited a relatively mild overall dysbiosis, with a structure closer to that of wild-type mice, consistent with the evolutionary pattern of microbiota during natural aging (decreases in *Akkermansia, Bifidobacterium, Faecalibaculum, Bacteroides*) (44, 45). Notably, although the high-fat diet induced dysbiosis, the resulting dysbiotic structure demonstrated a certain stability, with with only lower microbiota volatility compared to the normal and wild-type groups. This suggests that under sustained dietary intervention, the microbiota may evolve toward a new, structurally stable “dysbiotic steady state.” This finding provides a theoretical basis for reshaping the gut microecology through long-term, stable nutritional interventions to combat atherosclerosis.

At the metabolite level, this study identified two classes of key pathogenic metabolites: Diacylglycerols (DAG) and their homologs Dg(18:4(6Z,9Z,12Z,15Z)/18:1(11Z)/0:0), Dg(9D3/9M5/0:0), and Dg(20:4(8Z,11Z,14Z,17Z)/15:0/0:0), as well as the hydroxylated vitamin D metabolite 25-hydroxycholecalciferol, both of which were significantly elevated in the high-cholesterol group. DAG originates from various sources, including intermediate products generated by the enzymatic digestion of exogenous lipids in the intestine, as well as accumulation due to the activation of endogenous synthesis pathways in liver and intestinal cells (46, 47). Furthermore, bile acid metabolism alterations induced by microbiota dysbiosis and the impact of microbiota-derived metabolites on systemic inflammation and insulin sensitivity also contribute to DAG accumulation (48, 49). As a natural activator of Protein Kinase C (PKC), DAG can induce insulin resistance via the PKC signaling pathway and activate pro-inflammatory pathways such as NF-κB, thereby synergistically promoting atherosclerosis development at both metabolic and inflammatory levels (50, 51). Therefore, DAG can be considered a key nodal molecule connecting adverse diet, host metabolic disorders, and an imbalanced gut microenvironment.

On the other hand, the increase in 25-hydroxycholecalciferol might be related to disturbances in the enterohepatic circulation of bile acids caused by microbiota dysbiosis, affecting vitamin D absorption and hydroxylation processes (52, 53). At elevated levels, this metabolite may promote the transdifferentiation of vascular smooth muscle cells into osteoblast-like cells, driving the vascular calcification process, and participate in immune regulation disorders, thereby playing a role in plaque hardening and advanced lesions (54, 55). DAG and 25-hydroxycholecalciferol act synergistically from two different dimensions—metabolic inflammation and vascular structural remodeling—forming a harmful metabolic network that promotes atherosclerosis development.

In summary, this study demonstrates that although genetic background plays a role in the onset of atherosclerosis, environmental factors such as diet exert a significant, potentially even dominant, influence on disease progression. This suggests that adjusting the overall dietary structure and controlling key metabolic and inflammatory risk factors to restore the body's compensatory capacity may effectively delay the disease process. Additionally, in the state of chronic inflammation, the decrease in LDL sialylation, particularly more pronounced in the high-cholesterol group, warrants further investigation into its potential as an early biomarker for atherosclerosis and deeper exploration of mechanisms other than inflammation that might cause LDL desialylation. Scientifically guided nutritional interventions to modulate gut microbiota structure, maintain normal lipoprotein modification states, and block the accumulation of pathogenic metabolites may become effective strategies for preventing and treating atherosclerosis.

## 5 Conclusion

Under the combined pressure of a high-cholesterol dietary challenge and LDLR knockout, atherosclerosis progression is markedly accelerated, exceeding what can be attributed to genetic susceptibility alone. This process appears to be driven by diet-induced gut microbiota dysbiosis, characterized by a structural shift marked by the enrichment of pathobionts and depletion of beneficial commensals, together with broad functional disruption involving energy metabolism, cellular structural biosynthesis, and genetic information-processing pathways. This coordinated structure-function dysregulation may amplify systemic chronic inflammation through a microbiota-metabolism-immune axis, promote the accumulation of pathogenic metabolites, enhance LDL desialylation, and thereby accelerate atherogenesis. Notably, although high-cholesterol feeding induces pronounced microbial dysbiosis, it also establishes a relatively stable dysbiotic steady state, suggesting that long-term dietary modulation may provide an opportunity to reshape the gut microecology and modify atherosclerosis risk.

## Data Availability

The raw sequencing data from this study have been deposited in the Genome Sequence Archive (GSA) at the BIG Data Center (https://ngdc.cncb.ac.cn/gsub/), Beijing Institute of Genomics (BIG), Chinese Academy of Sciences, under the accession number CRA033722. Shared URL: https://ngdc.cncb.ac.cn/gsa/s/bEE0nRvp.
