# Supplementary figures and images for: Temporal gut microbiota dysbiosis links metabolic impairment, LDL desialylation, and accelerated atherosclerosis in LDLR−/− mice
Source: Front Med (Lausanne). 2026 Jul 1;13:1754833. doi: 10.3389/fmed.2026.1754833 (PMC13372019; doi:10.3389/fmed.2026.1754833)

(A)

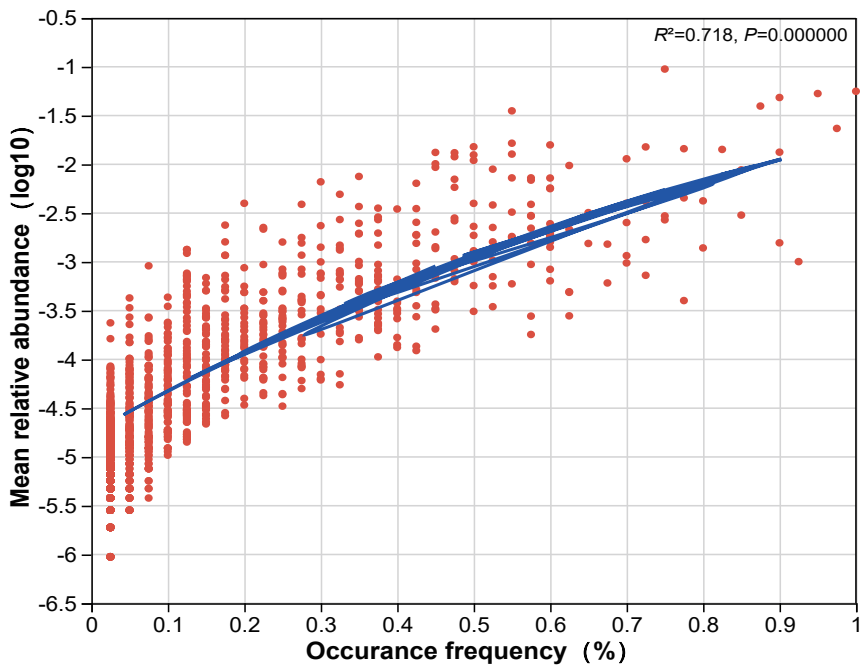

(B)

Correlation Heatmap

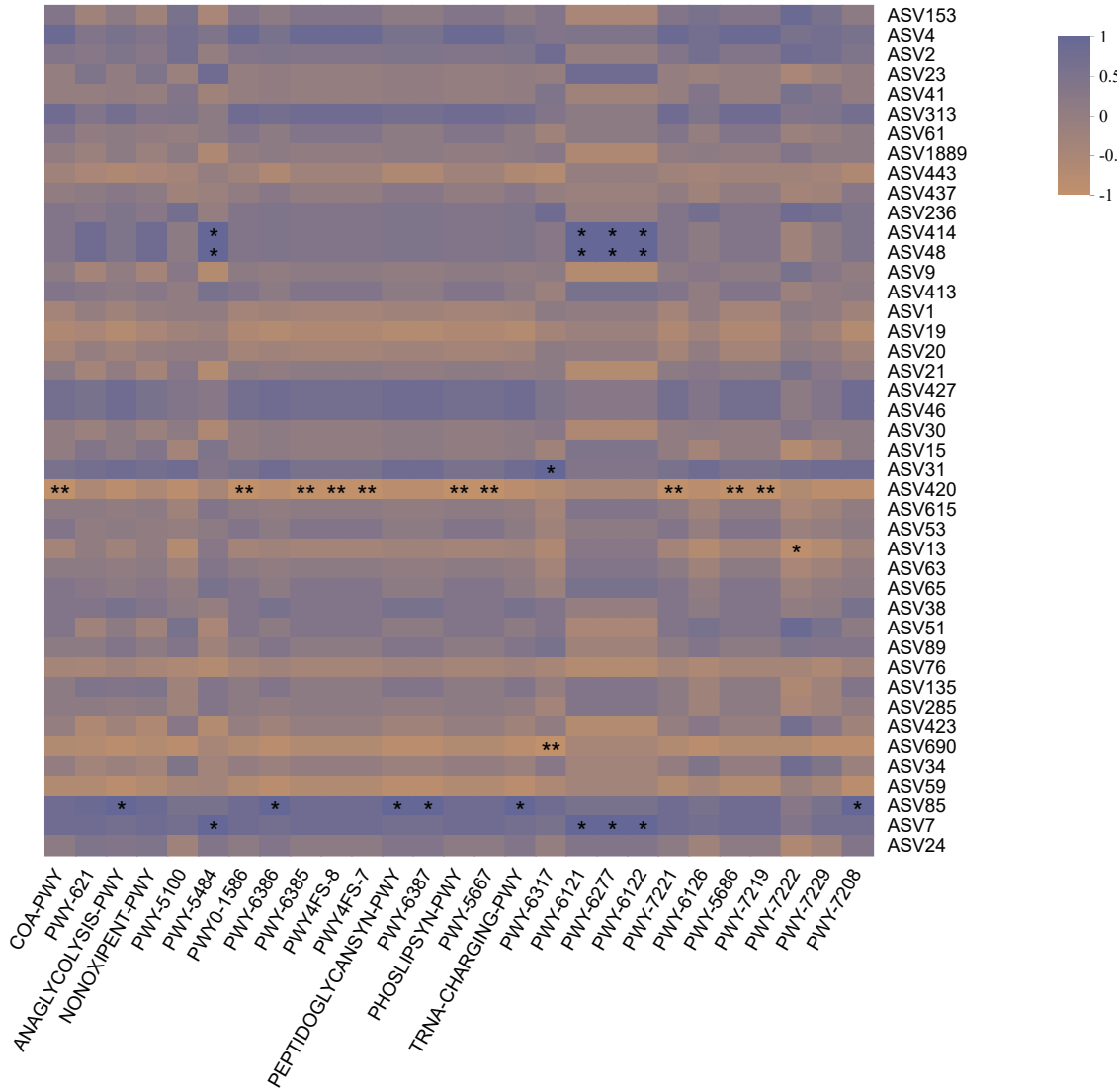

(D)

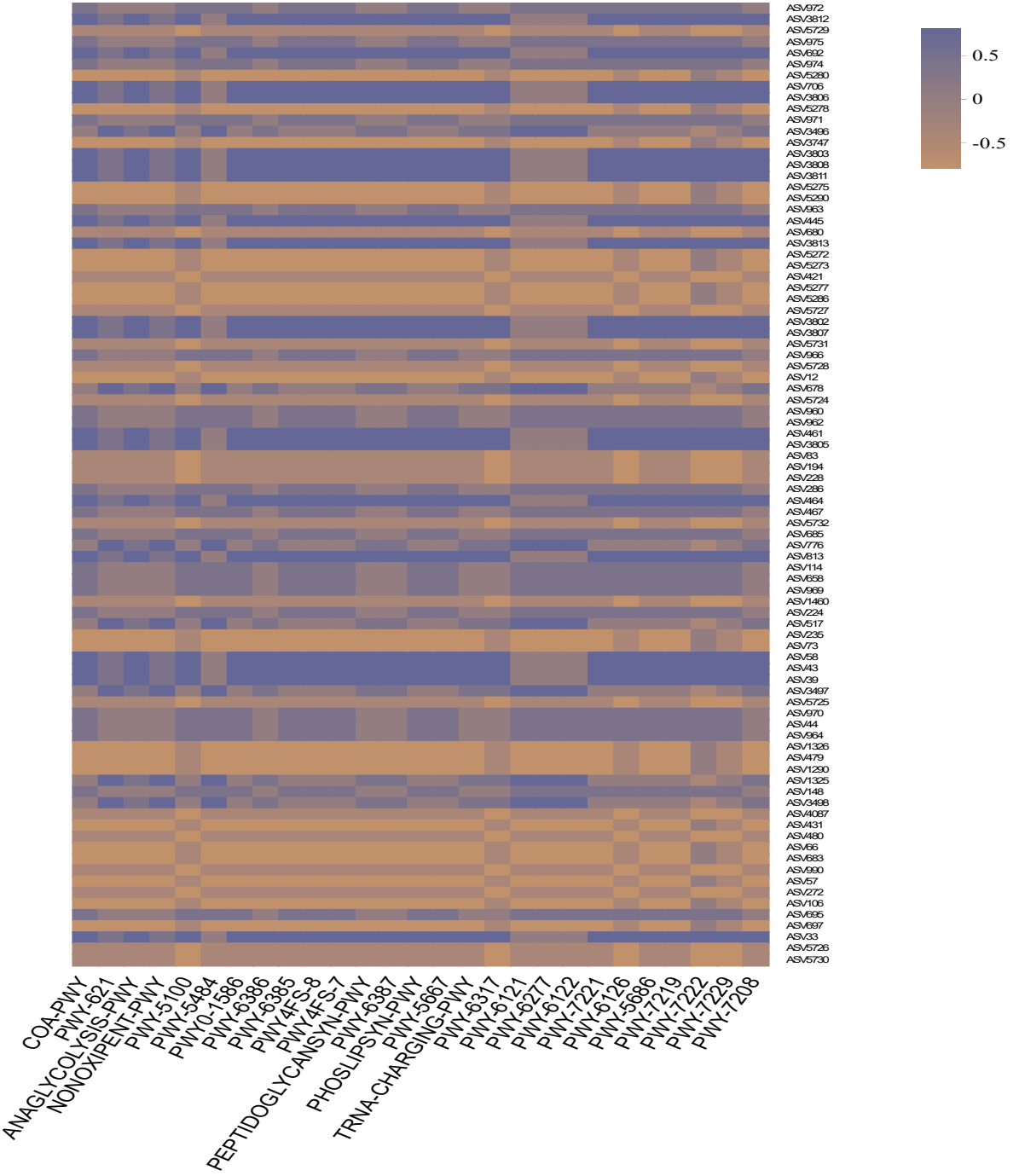

(C)

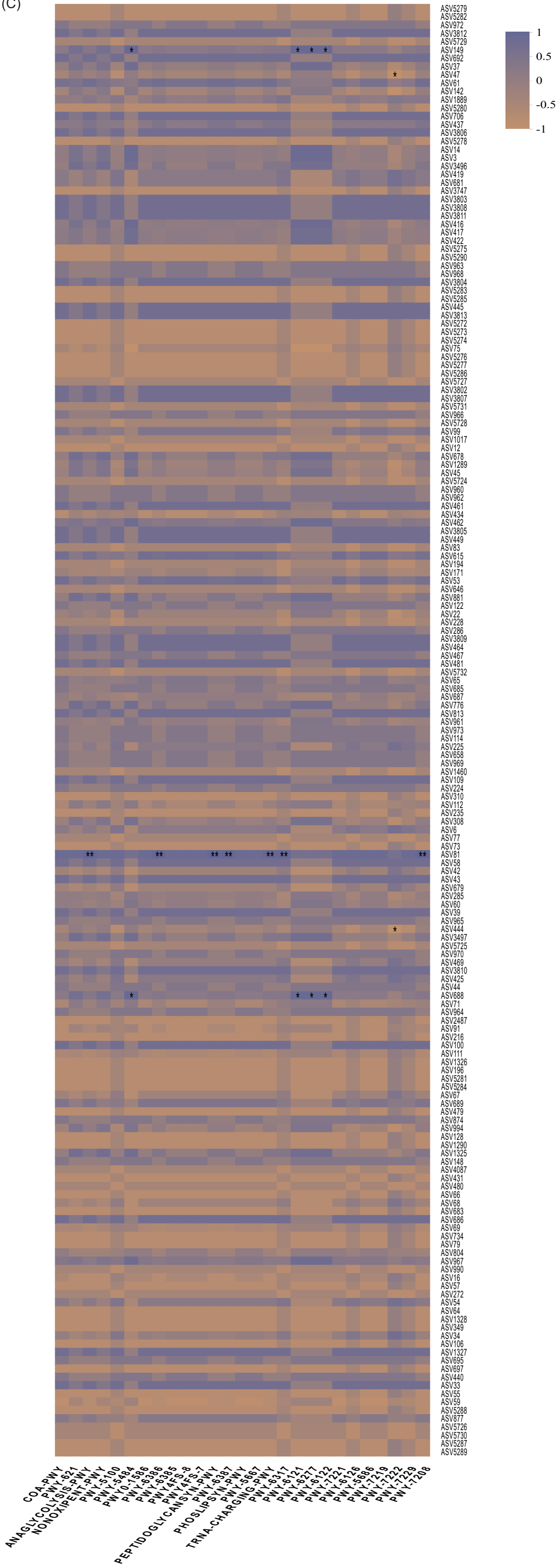

Supplement: Supplementary file 1 [file Data_Sheet_1.pdf]
