# Supplementary material for: Temporal gut microbiota dysbiosis links metabolic impairment, LDL desialylation, and accelerated atherosclerosis in LDLR−/− mice
Source: Front Med (Lausanne). 2026 Jul 1;13:1754833. doi: 10.3389/fmed.2026.1754833 (PMC13372019; doi:10.3389/fmed.2026.1754833)
Supplement: Supplementary file 3 [file Table_1.docx]

Table S1 Calibration curve data for sialic acid standards

| Concentration (ng/mL) | Area |
| --- | --- |
| 1000 | 785140 |
| 500 | 389777 |
| 200 | 157031 |
| 100 | 79026 |
| 50 | 38579 |
| 20 | 13828 |
| 10 | 5576 |

*Note: Linear regression equation: y = 784.036x + 0.000, coefficient of determination *R*² = 0.9999.The mean response factor (RF) was 737.24 with an RF %RSD of 11.71%, indicating good response consistency across the calibration range*.
